# DEVELOPMENT AND PSYCHOMETRIC TESTING OF THE PERSON-PLACE FIT MEASURE FOR OLDER ADULTS

**DOI:** 10.1093/geroni/igz038.2238

**Published:** 2019-11-08

**Authors:** Joyce Weil

**Affiliations:** University of Northern Colorado, Colorado, United States

## Abstract

Person-environment fit models are at the core of gerontological theory and practice. The Person-Place Fit Measure for Older Adults (PPFM-OA) was developed to capture key place domains arising from the expansion of aging in place and age-friendly concepts across the continuum of care, increasingly diverse older populations, and the changing meaning of place. The goal of this study was to create a validated measure to assess “fit”. Data from in-depth interviews, focus groups, review of literature and existing comparable measures, and cognitive interviewing created an initial instrument of 90 items. Through the use of a Delphi panel of experts, 60 items were selected and tested in the final measure on Mechanical Turk (MTurk). Results from a sample of persons 60 and older are discussed. Findings from psychometric testing of the PPFM-OA measure are reported. These findings show the final, further-reduced-item version of the Person-Place Fit Measure for Older Adults has good internal consistency and validity. Both this measure and its development process can advance understanding and measurement of place models.

